# SARS-COV-2 and Ocular Surface: From Physiology to Pathology, a Route to Understand Transmission and Disease

**DOI:** 10.3389/fphys.2021.612319

**Published:** 2021-02-12

**Authors:** Dalton de Freitas Santoro, Luciene Barbosa de Sousa, Niels O. S. Câmara, Denise de Freitas, Lauro Augusto de Oliveira

**Affiliations:** ^1^Department of Ophthalmology and Visual Science, Federal University of São Paulo, São Paulo, Brazil; ^2^Department of Immunology, Institute of Biomedical Sciences IV, University of São Paulo, São Paulo, Brazil; ^3^Nephrology Division, Federal University of São Paulo, São Paulo, Brazil

**Keywords:** COVID-19, SARS-COV-2, ocular surface, ACE2 receptor, conjunctiva, IgA, tear film, lactoferrin

## Abstract

Coronaviruses gained public attention during the severe acute respiratory syndrome (SARS) outbreak in East Asia in 2003 and spread of Middle Eastern respiratory syndrome (MERS) in 2012. Direct human-to-human contact and droplet are the main methods of transmission. Viral stability in aerosols on different surfaces supports evidence on indirect viral acquisition from fomites through the mucous membranes of the mouth, nose, and eyes. Given the pandemic circumstances, the level of evidence in COVID-19 and ophthalmology regarding eye infection, conjunctival transmission, and viral shedding through tears is insufficient. Presently, conjunctival transmission of coronaviruses has not been confirmed and remains controversial. Considering the physiology of the lacrimal system and ocular surface, the eyes are considered an immunoprotective site, with several antiviral molecules and anti-inflammatory proteins. Nevertheless, they represent an interface with the exterior world and face daily putative aggressors. Understanding the host’s ocular surface immunological and protective environment is crucial to clarify the potential of the conjunctiva as an entry route for SARS-CoV-2 and as part of this viral infection. We will discuss hypothetical ocular surface transmission mechanisms and related counterarguments addressed to both angiotensin-converting enzyme 2 receptors found on the conjunctival and corneal epithelia and lactoferrin, lysozyme, lipocalin and secretory IgA levels in the tear film. Hopefully, we will promote better understanding of this organ in COVID-19 infection and the potential transmission route that can be helpful in setting recommendations on best practices and protective guidelines to mitigate the disease spread.

## Introduction

In December 2019, a new type of respiratory disease emerged in China, specifically in Wuhan province, with several reports of new daily cases showing that the new disease was rapidly spreading. On January 30, 2020, the World Health Organization (WHO) declared the new disease, named coronavirus disease 2019 (COVID-19), a public health emergency of international interest and, on March 11, 2020, a pandemic. Three months after the first case and declaration of a pandemic condition, the world saw the power of this new virus, named SARS-CoV-2, belonging to an already known family of coronaviruses. Although SARS-CoV-2 has similarities to SARS-CoV, the causative agent of SARS in 2003, and MERS-CoV, the causative agent of MERS in 2012, it seems to be more effective in its transmission. To date, the number of infected individuals worldwide is 56.684.638, and the total number of deaths is 1.356.365 (COVID-19 dashboard by the Center for Systems Science and Engineering (CSSE) at Johns Hopkins University (JHU), 2020).

Therefore, understanding the aspects related to the transmission of the virus becomes crucial to contain the rapid spread of the disease and propose adequate safety protocols for the protection of health professionals and the entire population against COVID-19. The coronavirus family is known for transmission through contact with infected individuals, through the inhalation of droplets and aerosols expelled by them. However, since the transmission of SARS-CoV-2 is much higher, given its rapid dissemination to all continents, new and unclear forms of transmission need to be investigated. Likewise, understanding the peculiarities of the new virus and host invasion mechanisms are of paramount importance.

On January 22, 2020, a respiratory disease specialist from the Joint Commission of the WHO on a mission in Wuhan declared that he contracted COVID-19, even though he wore an N95 mask and gloves but did not wear goggles, starting the disease with symptoms of conjunctivitis, increasing the risk of ocular transmission of SARS-CoV-2 (Dai, 2020). The ocular surface, being exposed to aerosols and droplets, could be the route of transmission of the new coronavirus through direct penetration of the virus into the epithelial cells of the conjunctiva and cornea. Additionally, through the nasolacrimal drainage system, the virus could be drained by the tear film through the nasolacrimal duct to the upper airways, initiating transmission.

This review aimed to discuss hypothetical ocular surface transmission mechanisms based on known pathogenic requirements by the virus to penetrate host cells and related counterarguments addressed to angiotensin-converting enzyme 2 (ACE2) receptors found on the conjunctival and corneal epithelia as well as to lactoferrin, secretory IgA, lysozyme and lipocalin levels in the tear film.

## Coronavirus Family

The coronavirus family has three subtypes already identified: alpha, beta, and gamma. Alpha and beta subtypes are causative agents of diseases in mammals, while gamma subtype affects birds (Fenner et al., 1974). Two alpha coronaviruses are related to human diseases: human coronavirus 229E and human coronavirus NL63. From the beta subtype, human coronavirus HKU1, human coronavirus OC43 (the most common of all human coronaviruses), SARS-CoV, MERS-CoV, and SARS-CoV-2 are known to be the causative agents of human diseases (Fenner et al., 1974).

A common anatomical characteristic of this family of viruses is the lipid membrane that envelops and confers its structure. Besides that, all coronaviruses have structural proteins, non-structural proteins, and accessory membrane proteins. The spike protein is attached to the envelope of the virus and represents the key element to bind with the host receptor. They are single-stranded RNA viruses, and their name is derived from the resemblance to a crown that the spike proteins confer on the surface of the virus (Wertheim et al., 2013).

Understanding the diseases caused by this family of viruses to animals might be helpful in elucidating the behavior and mutation ability of those that affect humans. For example, in rats, there are two distinct, well-defined biotypes: a variant that causes mainly gastrointestinal diseases and another that affects multiple organs. In cats, feline coronaviruses (FeCoVs) are less aggressive and present tropism to the apical epithelium of the intestine. However, 5% of the cases will develop peritonitis, a serious disease with increased mortality. FeCoVs are believed to mutate, becoming feline infectious peritonitis viruses (FIPVs) that cause vasculitis. Ocular findings from cats contaminated with FIPV resemble cases of vasculitis, such as granulomatous uveitis, choroiditis, and retinal detachment. High culture growth rate (90%), including viable virus identification, from conjunctival samples suggest the risk of ocular transmission (Willcox et al., 2020). Thus, the possibility of ocular involvement in humans and viral transmission through this route must be considered.

Among all coronaviruses that affect humans, HCoV NL63 caused conjunctivitis in children in 17% of cases (Seah and Agrawal, 2020). SARS was the first pandemic of the twenty-first century, in 2004, and no ocular involvement was reported (LeDuc and Barry, 2004); however, Loon et al. (2004) demonstrated the presence of viral particles by conjunctival polymerase chain reaction (PCR) even without associated conjunctivitis. During the MERS outbreak, there were also no reports of ocular involvement (Loon et al., 2004).

Genetic studies have shown that SARS-CoV-2 has 90% genetic similarity to the bat’s coronavirus (CoV RATG13), suggesting that they might be the natural reservoir for these viruses and that COVID-19 is possibly a zoonosis (Zhou P. et al., 2020). Mackenzie and Smith (2020) and Guo et al. (2020) found 79% genetic similarity between SARS-CoV-2 and SARS-CoV. Lu et al. (2020) found 50% similarity between MERS-CoV and SARS-CoV-2. Thus, knowledge acquired from previous epidemics are guiding current investigations to elucidate SARS-CoV-2 potential similarity in pathophysiological mechanisms of penetration into host cells and its increased transmissibility.

## Renin-Angiotensin System and Ocular Surface Area

SARS-CoV-2 needs the ACE2 receptor to be able to invade host cells, like SARS-CoV (Hoffmann et al., 2020; Lan et al., 2020). The renin-angiotensin system (RAS) is an important modulator of the volume of body fluid and directly involved in the regulation of systemic blood pressure. Angiotensin is produced by the liver and converted into angiotensin 1 by renin. Subsequently, angiotensin 1 is converted into angiotensin 2 by ACE. Angiotensin 2 binds to various ACE receptors (subtypes 1 and 2) distributed in various tissues. There is also local RAS tissue that can locally produce various components of the renin-angiotensin system but has specific functions in each organ. The RAS tissue has already been identified in the heart, lung, reproductive system, central nervous system, gastrointestinal system, breast, pancreas, and adrenal and eye tissues (Yaguchi et al., 2012).

The ocular RAS components have been identified in the cornea, conjunctiva, sclera, ciliary body, retina, aqueous humor, vitreous humor, and iris (Yaguchi et al., 2012; Giese and Speth, 2014; Vaajanen et al., 2015). Danser et al. (1989) demonstrated the inability of the renin-angiotensin plasma enzymes to penetrate into the eye, and Wagner et al. (1996) demonstrated the local production of the RAS components in ocular tissues and the importance of this system in the control of ocular physiology (Danser et al., 1989; Wagner et al., 1996). Ocular RAS has been linked to several eye diseases: diabetic retinopathy, glaucoma, age-related macular degeneration, uveitis, cataracts, and dry eye syndrome (Holappa et al., 2017).

As SARS-CoV-2 requires ACE2 receptors to penetrate host cells, the presence of ocular RAS raise the hypothesis of COVID-19 transmission through the ocular surface route.

## Ocular Surface Route to SARS-CoV-2

The ocular surface, formed by epithelial cells of the cornea and conjunctiva, is exposed to the environment and, therefore, can be contaminated by droplets and aerosols of individuals infected with SARS-CoV-2 or even by contact with contaminated fomites. Additionally, the ocular surface is connected to the upper airways via the nasolacrimal duct and although less likely could receive viruses ascending through this path (Willcox et al., 2020).

Interestingly, during the influenza virus epidemic of 1919, Maxcy hypothesized that the ocular surface, which presents a surface area larger than that of the nostrils and oral cavity, would be more prone to infection by contaminated particles (Maxcy, 1919). Moreover, after comparing the ocular surface area not exposed during blinking and the area not exposed during mouth closure, Coroneo MT found that the surface of the conjunctiva and cornea was noticeably more exposed (Coroneo, 2021). However, it is also important to note that both the oral cavity and nostrils act as a continuous aerosol aspiration pump during inspiration, whereas the ocular surface has no such function and instead requires particles to be directly introduced through aerosols sprayed against it.

**FIGURE 1:**
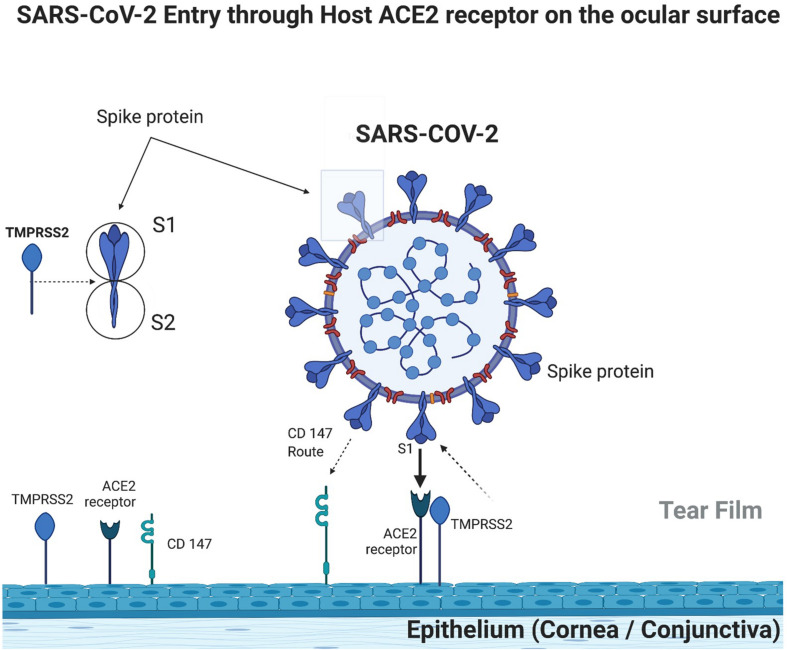
Ocular surface immunobiology and SARS-COV-2 entry mechanism (created with BioRender.com).

SARS-CoV-2 requires the presence of ACE2 receptors to penetrate host cells, which occurs through the binding of viral spike protein to the host’s ACE2 receptor. It has also been demonstrated that a protease on the host cell surface, TMPRSS2, also known as Furin protein, promotes the priming of the S subunit of the spike protein in S1, which is related to the binding of the ACE receptor, and S2, which is related to the fusion of the virus lipid membrane with the cell membrane (Hoffmann et al., 2020). Without TMPRSS2, the virus can penetrate host cells via endocytosis but in a much less efficient way (Matsuyama et al., 2005; Figure 1).

A few studies already demonstrated ACE2 receptors in the cornea and conjunctiva, which in theory accomplish SARS-CoV-2 requirement to invade host cells in the ocular surface. Zhou L. et al. (2020) demonstrated the positivity of ACE2 receptors in the corneal epithelium and endothelium, although absent in the corneal stroma. They showed a more expressive positivity in the limbus than in the superficial cells of the cornea (Zhou L. et al., 2020). Such findings were also observed by Ma et al. (2020). Collin et al. (2021) also found positive ACE2 receptors in the corneal epithelium, with higher expression in the limbus. Zhou L. et al. (2020) demonstrated the positivity of ACE2 receptors in the conjunctiva, mainly in the superficial cells, but absent in goblet cells and rarely found below the epithelium, in the substantia propria. Ma et al. (2020) found greater expression of ACE2 receptors in the conjunctiva than in the cornea, suggesting greater probability of SARS-CoV-2 penetration in the conjunctiva rather than in the cornea. Likewise, Leonardi et al. (2020) also found positivity for ACE2 receptors in the conjunctiva in low expression but greater than in the cornea.

As cellular invasion is facilitated by the protease TMPRSS 2 (Furin), its expression in ocular tissues is important to evaluate the ocular surface as a route of infection by SARS-CoV-2. Zhou L. et al. (2020) found strong positivity of the Furin protease in the corneal epithelium and endothelium; however, unlike ACE2 receptors, it was also found in the corneal stroma and all limbus layers. Collin et al. (2021) also demonstrated positivity of Furin protease in the central and peripheral corneal epithelium and the limbus.

Zhou L. et al. (2020) found strong positivity of Furin protease in conjunctival epithelial cells and weak positivity in the stroma, being absent in goblet cells. Conversely, Ma et al. (2020) found extremely low expression of Furin protease in the conjunctiva in relation to the cornea, suggesting that invasion more likely occurs in the corneal epithelium rather than in the conjunctiva. Leonardi et al. (2020) found expression of TMPRSS2 protease only in the conjunctiva but not in the cornea. Collin et al. (2021) found positivity with high expression in the superficial and basal conjunctival epithelium.

Again, both the cornea and conjunctiva with TMPRSS protease (Furin) expression show the favorable elements for the invasion by SARS-CoV-2, thus making this transmission route possible.

Although there are similarities between SARS-CoV-2 and SARS-CoV, it is known that SARS-CoV-2 binds more avidly to ACE2 receptors. So far, this suggests the role of other factors involved in SARS-CoV-2 adhesion efficiency. Wang et al. (2020) demonstrated that SARS-CoV-2 invades host cells through a new route, using CD147, which is a transmembrane glycoprotein from the immunoglobulin family. Leonardi et al. (2020) found positivity in intermediate CD147 levels in both the cornea and conjunctiva. Thus, SARS-CoV-2 could invade the ocular surface through this transmembrane glycoprotein (CD147) route (Figure 1).

## COVID-19 and Ocular Manifestations

It is interesting that the ocular surface accomplishes known requirements for SARS-CoV-2 invasion but local viral detection and ocular manifestations in patients diagnosed with COVID-19 are rare. This may not be a preferred site of viral entry and neither for its replication. Several studies have searched for viral particles in the conjunctiva (conjunctival swabs) and tear film (Schirmer strips, microcapillary tubes, and tear washing) using molecular techniques and mostly reported low and variable positivity. Sarma et al. (2020) conducted a meta-analysis and reported that the positivity of the conjunctiva or tear samples was 1.95% using molecular techniques (PCR). The authors discussed that the low positivity might be related to the date of sampling, at a time when the viral replication in the conjunctiva or tear may be absent, especially in more severe disease. Another issue discussed in a previous study regarding low positivity testing is the small amount of material obtained from both the conjunctiva and tear film. Worse yet, this small amount is usually diluted in buffer solution before running the molecular test. Zhang et al. (2020) found only one patient with conjunctival PCR positive for SARS-CoV-2 in 72 patients with confirmed diagnosis of COVID-19 by nasopharyngeal swab, who presented symptoms of conjunctivitis, suggesting that the positivity is greater when there is ocular involvement. Seah et al. (2020) conducted a viral shedding study using conjunctival swab in 17 patients with confirmed diagnosis of COVID-19 (total of 64 samples over the first, second, and third weeks after symptom onset). None of the patients had ocular symptoms, except one who developed hyperemia and chemosis during follow-up, and all analyzed samples were negative. Xie et al. (2020) analyzed conjunctival swab from 33 patients one week after systemic symptom onset, and none of the patients in this series presented ocular manifestations, and in two patients, the conjunctival sample showed a positive result. Wu et al. (2020) reported that 2 of 11 patients with ocular manifestations and confirmed diagnosis of COVID-19 by nasopharyngeal swab presented positive PCR in the conjunctival swab, suggesting that, even in patients with ocular alterations, the positivity is low.

Regarding ocular clinical findings, Sarma et al. (2020) found follicular conjunctivitis, which is common to other viral infections, in 3.17% of all patients confirmed with COVID-19 and 0.7% of these patients presented ocular manifestation as the first symptom. Other symptoms reported in this meta-analysis were conjunctival congestion (3.8%), foreign body sensation (19%), excessive tearing (13.3%), eye pain (5.7%) and increased conjunctival secretion (3.8%). To date, there are no studies on visual loss in any patient diagnosed with COVID-19 (Sarma et al., 2020).

Torres-Costa et al. hypothetically suggested that SARS-CoV-2 may have a neurotropism common to the coronavirus family and therefore affect ocular neurological structures (Torres-Costa et al., 2020). In fact, Marinho et al. (2020) found retinal changes in 12 patients with confirmed diagnosis of COVID-19 (nasopharyngeal PCR and antibody detection) using optical coherence tomography to analyze the retinal layers. All patients showed hyper-refringent lesions at the level of the ganglion cells and inner plexiform layer, mainly in the papillary-macular bundle, but without visual alteration, which suggests the possibility of neurological involvement (Marinho et al., 2020).

Colavita et al. (2020) found positive samples of conjunctival PCR in the first patient diagnosed with COVID-19 in Italy, presenting bilateral follicular conjunctivitis since the onset of systemic symptoms. Interestingly, the PCR from the conjunctival samples showed positive results until 21 days after disease onset, with a progressive decrease in the viral concentration over time. Moreover, to prove viable viruses with ocular transmission, the material collected from the first positive sample was inoculated into Vero E6 cells, and there was viral replication confirmed by PCR, suggesting the potential risk of infection through the eye.

Regarding ophthalmologists’ exposure and their potential role as spreaders, Coroneo MT determined that a proximity of approximately 38 cm between ophthalmologists and patients during slit lamp exam would place them at risk for transmission given that viral particles could easily travel through aerosols expelled during breathing and speech at distances of 30 cm (Coroneo, 2021). Rokohl et al. (2020) also expressed their concern regarding the need for ophthalmologic offices to adopt protective measures, such as protective barriers in the slit lamp and mandatory disinfection of all ophthalmic equipment coming into direct contact with patients at the end of each medical appointment, to mitigate SARS-COV-2 spread.

## Ocular Surface Immunological and Protective Environment Against Viral Infection

A healthy human tear film provides oxygen and nutrients and contains a series of proteins that together provide a protective barrier against the invasion of various pathogens. In this scenario, a few components stand out and will be discussed: lactoferrin, immunoglobulin A (IgA), lysozyme, and lipocalin. We will also discuss in this section the tear film clearance associated to blinking rate and amount of ACE2 receptors in the ocular surface compared to other organs.

Lactoferrin, first described by Masson et al. (1966), gained notorious importance for its anti-inflammatory, antibacterial, and antiviral actions. It is a mammalian glycoprotein secreted by the exocrine glands and neutrophils. One of its immunoprotective property is associated with iron chelation, which results in hololactoferrin. Iron has an important role in cellular oxidation-reduction reactions and oxygen transport. It is also essential in DNA replication and energy generation. Thus, it is crucial for the microorganism’s survival and replication. Lactoferrin acts as an anti-inflammatory agent by sequestering iron molecules (Kell et al., 2020).

Lactoferrin’s antiviral action has already been proven in relation to human immunodeficiency virus (HIV), rotavirus, herpes simplex virus, and human papillomavirus (Kell et al., 2020). A few mechanisms are involved in its antiviral response: direct competition to receptors used by viruses for cell invasion; direct linkage to some viral particles; and ability to bind to heparan sulfate, which serves as the first viral anchorage site in cell membranes and facilitates cell invasion by pathogens. Lang et al. (2011) demonstrated that lactoferrin markedly inhibits the invasion of SARS-CoV pseudovirus (Lang et al., 2011) probably by binding to heparan sulfate, which serves as a link to sites of low anchoring of the SARS-CoV to the ACE2 receptor (Lang et al., 2011). As SARS-CoV and SARS-CoV-2 present an increased genetic similarity (75%) and use ACE2 receptors, it seems reasonable that heparan sulfate could work as the first SARS-CoV-2 cell membrane contact. These corroborates that lactoferrin, which is normally found in the tear film, presents an important antiviral mechanism acting as a barrier to the invasion of this virus through the eye.

Regarding IgA, this immunoglobulin is largely distributed in the mucous membranes in the body and is the most abundant immunoglobulin in the human tear film. Lacrimal IgA is mainly produced by the lacrimal gland, and its concentration rapidly increases in the presence of pathogens that attack the ocular surface. Its protective action has already been proven against viruses by preventing its binding to host’s cell receptor, against bacteria by preventing its attachment and colonization, and against amoebae (O’Sullivan and Montgomery, 2015). The IgA antibody binds to SARS-CoV-2 spike protein, blocking its interaction with the host’s ACE2 receptor, thus preventing the virus from entering human cells. IgA seroconversion in patients with COVID-19 can already be detected after 2 days of infection, and after the first month of infection, the seroconversion positivity is approximately 100% (Yu et al., 2020). With this background, IgA in the tear film is another important defense mechanism against aggression to the ocular surface, making it less likely that SARS-CoV-2 will invade the eye. Yu et al. (2020) demonstrated the highest serum positivity of IgA antibody in patients with severe COVID-19, suggesting the possibility of an immune-mediated disease through deposition of IgA and vasculitis.

Lysozyme, discovered by Alexander Flemming in 1922 (Tan and Tatsumura, 2015), is produced by the accessory tear glands and has already proven its activity against bacteria (Masschalck and Michiels, 2003), viruses (Lee-Huang et al., 1999), and fungi (Samaranayake et al., 2001). Ferrari et al. (1959) reported the effects of lysozyme against several viruses known at that time as herpes simplex and herpes zoster (Ferrari et al., 1959). Lee-Huang et al. (1999) demonstrated the inhibition of HIV replication by lysozyme. The effectiveness of this enzyme against SARS-CoV-2 has not yet been demonstrated, and further studies are needed to elucidate whether it can also act as a barrier to the entrance of SARS-CoV-2 through the ocular surface. However, hypothetically, it is likely that its presence in the tear film has some effect on blocking the entrance of SARS-CoV-2 through the cornea and conjunctiva.

**FIGURE 2:**
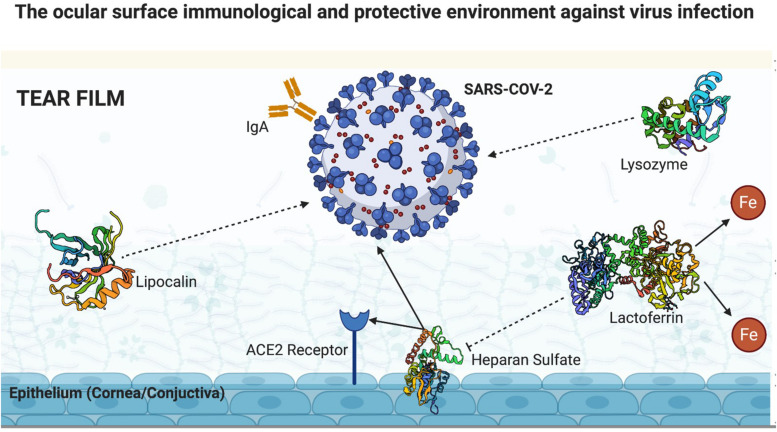
Ocular surface immunological and protective environment against virus infection (created with BioRender.com).

Lipocalins are a family of diverse, low molecular weight proteins that predominantly function extracellularly. Tear lipocalin, first reported by Erickson in 1956 (Erickson, 1956), is present in large quantities in tear film, although not a tear-specific protein. Flower (1996) reported a few common characteristics of these proteins, including (1) their ability to bind to smaller, mainly hydrophobic, molecules, (2) their ability bind to specific receptors on the surface of cells, and (3) the formation of macro-molecular complexes (Flower, 1996). Tear lipocalin is a multitasking protein whose functions and receptors are yet to be fully elucidated. Dartt reported a few already identified functions, which include participating in the regulation of the immune system, modulation of cell growth, and metabolism of apolipoprotein-D (Dartt, 2011). Moreover, lipocalin possesses antimicrobial activity that interferes with microbial siderophores, thereby decreasing the absorption of iron essential for their replication. Furthermore, it has a proven antibacterial and antifungal activity, and a potential antiviral action as well (Fluckinger et al., 2004). Nonetheless, further studies are needed to determine whether lipocalin can also interfere with SARS-COV-2 invasion of the ocular surface (Figure 2).

The act of blinking also plays a role as a protective mechanism against the invasion of pathogens in the ocular surface. Tsubota et al. reported that the human blink rate under relaxed conditions is up to 22 blinks/min (Tsubota and Nakamori, 1993). Blinking provides a continuous washing mechanism for microorganisms on the ocular surface, clearing them by the lacrimal duct (McClellan, 1997). Thus, a normal blink rate also decreases invasion of SARS-CoV-2 through the ocular surface. The retention time of the particles that penetrate the tear film has been related to its turnover rate, which depends on several factors, such as blink rate per minute, tear volume, climatic conditions (e.g., air humidity, environmental temperature, and wind), the use of contact lenses, and exposure time to digital screens. Local conditions (e.g., Meibomian gland dysfunction), systemic conditions (e.g., rheumatological diseases) affecting tear production, and systemic medications leading to decreased tear production can also interfere with tear film turnover and the retention time of viral particles within the tear (Pearce et al., 2011; Rolando et al., 2018). As a counterargument, since SARS-CoV-2 is mostly transmitted through the airways, the act of blinking itself could work as a pump propelling the viruses that are on the surface of the cornea and conjunctiva to the upper airways, through the nasolacrimal duct, thus allowing its transmission through the eye.

The tear film is drained through the tear punctum by continuous blinking, with the drainage occurring through the lower tear punctum. Tear drainage occurs through absorption mostly by the nasolacrimal drainage system (including tear ducts and the nasolacrimal gland until it reaches the upper airways) and to a lesser extent by the cornea and conjunctiva. However, a certain amount of tear film is also absorbed by the nasolacrimal duct tissue. Paulsen et al. (1998) described the entire drainage system of the tear film through the lacrimal canaliculi, lacrimal sac, and nasolacrimal ducts using histological, immunohistochemical, and electron microscopic assays. The authors demonstrated the presence of isolated goblet cells integrated into the epithelial cells of the lacrimal canaliculi and the presence of grouped goblet cells in the lacrimal sac, forming mucous glands (Paulsen et al., 1998). Goblet cells are known to produce mucin, a glycoprotein that not only lubricates and hydrates the mucosa of the tear drainage system but also functions as a barrier against harmful agents (Willcox et al., 2017). Paulsen et al. (1998) also demonstrated the presence of IgA throughout the tear drainage system, an allied antibody in the mucosal defense system that acts as a protective factor against pathogen invasion (Paulsen et al., 1998). Therefore, although the tear drainage system through the lacrimal canaliculi, lacrimal sac, and nasolacrimal duct can be contaminated by viral particles present in the tear film, a relevant mucosal defense system, mainly consisting of mucin and IgA that act as barriers preventing invasion through the epithelium of this drainage system, does exist. In addition, Ayub et al. (2003) demonstrated that the cavernous body of the lacrimal sac and lacrimal duct interferes in the tear drainage system through an autonomous response that is integrated to a complex innervation network with the cornea. Thus, an injured cornea (e.g., foreign body, inflammation, and infection) would increase both tear production and its outflow, consequently and actively draining harmful materials and protecting the ocular surface (Ayub et al., 2003).

Another factor that also deserves attention while investigating the ocular surface as a possible transmission route for SARS-CoV-2 is the lower ACE2 receptor expression on the cornea and conjunctiva epithelia in relation to other tissues, such as the lung and heart (Willcox et al., 2020). Thus, by lowering the requirements necessary for viral invasion, the ocular surface becomes less prone as a preferred route.

Miner et al. (2020) have just published a paper reporting another corneal immunological protection against viral infections. The authors demonstrated that type III interferon (IFN-l) and its receptor (IFNlR1) restrict herpes simplex virus 1 and Zika virus growth in the human cornea. Interestingly, they also demonstrated that human corneal tissue did not support SARS-COV-2 infection, even after blockade of the type III IFN receptor (Miner et al., 2020). Thus, supporting the fact that the ocular surface is less prone to viral invasion and replication.

## Lessons on Adenoviral Conjunctivitis and SARS- and MERS-Associated Conjunctivitis

Knowledge on the pathophysiological mechanisms involved in other ocular viral infections, specially conjunctivitis, could contribute to a better understanding of the potentially pathological interaction between SARS-CoV-2 and the ocular surface. Adenovirus conjunctivitis (DNA virus) is the most common viral infection in the ocular surface. Conjunctivitis caused by adenovirus is usually a self-limiting condition. Clinical features can vary from sporadic manifestations, such as pharyngoconjunctival fever (most prevalent strains 3, 4, and 7) to epidemic outbreaks, such as epidemic keratoconjunctivitis (most prevalent strains 8, 19, 37, and 54) (Chigbu and Labib, 2018). Classically, but non-specifically, follicular conjunctival reaction, conjunctival congestion, foreign body sensation, excessive tearing, and preauricular adenomegaly are present. Interestingly, adenoviruses use several cellular receptors to bind to host cells (CAR, CD 46, and sialic acid interact with the fine-knob protein of the adenovirus) in the infection onset. Different adenovirus strains use distinct host’s cell receptors. The known mechanisms of cell invasion are the clathrin-mediated endocytosis, micropinocytosis, and caveolin-mediated pathway, and depending on some types of cells, the virus can use more than one pathway to penetrate the host cell (Pennington et al., 2019). The most used receptor by the majority of strains is the coxsackievirus-adenovirus receptor (except subtype B and some members of subtype D). After cellular invasion, adenoviruses integrate their replication machinery to the host cell DNA, consequently replicating and infecting more cells. Adenovirus infection generates an immune response in both the cornea and conjunctiva. Superficial corneal epithelial cells produce cytokines, which stimulate stromal keratocytes to release lymphocytes recruiting interleukins. The levels of lymphocytes, natural killer cells, and monocytes significantly increase in the conjunctiva during the acute phase of infection. Conjunctival epithelial cells and goblet cells produce mucins (MUC1, MUC4, MUC-5, and MUC16) and cytokines known to decrease adenoviral invasion (Pennington et al., 2019). Thus, adenoviruses’ pathophysiological mechanisms of ocular surface invasion are different from what is already known as SARS-COV-2 pathophysiological requirements to invade the ocular surface. Ocular manifestations of SARS-COV-2, although rare, when compared to those of adenoviruses are similarly non-specific (follicular conjunctival reaction, conjunctival congestion, foreign body sensation, and tearing).

Regarding knowledge on pathophysiological mechanisms of SARS-CoV and MERS-CoV pandemic, although they use the same ACE2 receptors that SARS-CoV-2 uses, we could not find reports on clinical manifestations of ocular surface involvement during the SARS and MERS pandemic even though the presence of viral particles has been demonstrated by conjunctival PCR during previous outbreaks in patients without conjunctivitis. However, SARS and MERS outbreaks have been helpful in allowing tailored investigation on host cell receptor requirements for COVID-19 invasion in different human tissues.

## Conclusion Remarks

The ocular surface gathers the essential and yet described elements for SARS-CoV-2 invasion through the eye. Molecular approaches have found viral particles in the tear film and conjunctiva. Associated ocular surface manifestations of conjunctivitis are non-specific and relatively rare during the current outbreak. Despite accomplishing required pathophysiological conditions for SARS-CoV-2 invasion, we discussed relevant protective mechanisms that make the eye a less likely route of infection and transmission of COVID-19. One cannot assure that the eye/ocular surface is a privileged organ strong enough against SARS-CoV-2, considering the conjunction of protective mechanisms discussed in this review. The level of evidence in COVID-19 and ophthalmology regarding eye infection, conjunctival transmission, and viral shedding through tears is still insufficient. Research on novel cell membrane molecules and receptors and other local immunoprotective mechanisms to clarify the potential of the conjunctiva acting as an entry route for SARS-CoV-2 are undertaken. Presently, conjunctival transmission of coronaviruses has not been confirmed and remains controversial. Nevertheless, recommendations on the best practices and protective guidelines to mitigate this disease propagation remain necessary, including intense and judicious care in the disinfection of all ophthalmic diagnostic equipment in direct contact with the ocular surface.

## Author Contributions

DFS: research design, data acquisition and/or research execution, and manuscript preparation. LS and DF: data analysis and/or interpretation and manuscript preparation. NC and LO: research design, data analysis and/or interpretation, and manuscript preparation. All authors contributed to the article and approved the submitted version.

## Conflict of Interest

The authors declare that the research was conducted in the absence of any commercial or financial relationships that could be construed as a potential conflict of interest. The handling editor declared a shared affiliation, though no other collaboration, with one of the authors NC at the time of review.
